# Virus-like particle vaccine displaying an external, membrane adjacent MUC16 epitope elicits ovarian cancer-reactive antibodies

**DOI:** 10.1186/s13048-023-01325-9

**Published:** 2024-01-16

**Authors:** Hsin-Fang Tu, Margaret Wong, Ssu-Hsueh Tseng, Nattha Ingavat, Pola Olczak, Kin Israel Notarte, Chien-fu Hung, Richard B.S. Roden

**Affiliations:** 1 Department of Pathology, Johns Hopkins University, Baltimore, MD 21287 USA; 2 Downstream Processing (DSP), Bioprocessing Technology Institute (BTI), Agency for Science, Technology, and Research (A*STAR), Singapore, 138632 Singapore; 3 Department of Oncology, Johns Hopkins University, Baltimore, MD 21287 USA; 4 Department of Gynecology and Obstetrics, Johns Hopkins University, Baltimore, MD 21287 USA

**Keywords:** MUC16, CA125, Antibody, Virus-like particle, Vaccination, Ovarian cancer

## Abstract

**Background:**

MUC16 is a heavily glycosylated cell surface mucin cleaved in the tumor microenvironment to shed CA125. CA125 is a serum biomarker expressed by > 95% of non-mucinous advanced stage epithelial ovarian cancers. MUC16/CA125 contributes to the evasion of anti-tumor immunity, peritoneal spread and promotes carcinogenesis; consequently, it has been targeted with antibody-based passive and active immunotherapy. However, vaccination against this self-antigen likely requires breaking B cell tolerance and may trigger autoimmune disease. Display of self-antigens on virus-like particles (VLPs), including those produced with human papillomavirus (HPV) L1, can efficiently break B cell tolerance.

**Results:**

A 20 aa juxta-membrane peptide of the murine MUC16 (mMUC16) or human MUC16 (hMUC16) ectodomain was displayed either via genetic insertion into an immunodominant loop of HPV16 L1-VLPs between residues 136/137, or by chemical coupling using malemide to cysteine sulfhydryl groups on their surface. Female mice were vaccinated intramuscularly three times with either DNA expressing L1-MUC16 fusions via electroporation, or with alum-formulated VLP chemically-coupled to MUC16 peptides. Both regimens were well tolerated, and elicited MUC16-specific serum IgG, although titers were higher in mice vaccinated with MUC16-coupled VLP on alum as compared to L1-MUC16 DNA vaccination. Antibody responses to mMUC16-targeted vaccination cross-reacted with hMUC16 peptide, and vice versa; both were reactive with the surface of CA125+ OVCAR3 cells, but not SKOV3 that lack detectable CA125 expression. Interestingly, vaccination of mice with mMUC16 peptide mixed with VLP and alum elicited mMUC16-specific IgG, implying VLPs provide robust T help and that coupling may not be required to break tolerance to this epitope.

**Conclusion:**

Vaccination with VLP displaying the 20 aa juxta-membrane MUC16 ectodomain, which includes the membrane proximal cleavage site, is likely to be well tolerated and induce IgG targeting ovarian cancer cells, even after CA125 is shed.

## Background

Ovarian cancer is the most lethal gynecologic cancer, and the American Cancer Society estimates that in 2023 about 19,710 women will receive a new diagnosis of ovarian cancer, and 13,270 women will die from ovarian cancer. Epithelial ovarian cancer is particularly insidious because the deep pelvic location of the ovaries renders them relatively inaccessible to physical examination and symptoms that prompt early diagnosis are often vague or absent. There is currently no screening test for reliable detection of ovarian carcinoma in its early stages and therefore the majority of women (58%) present with advanced disease at diagnosis, which is associated with 5-year survival rates of 27% for stage III and 13% for stage IV. While most cases of ovarian cancer are spontaneous, a significant fraction are associated with heritable risk, e.g. ~15% have germline BRCA1 or BRCA2 mutations [1].

Women with germline predisposing mutations may opt for prophylactic bilateral salpingo-oophorectomy to reduce risk of ovarian cancer; however, this causes loss of fertility and does not prevent primary peritoneal carcinoma. Standard of care treatment for epithelial ovarian cancer includes total abdominal hysterectomy and aggressive surgical de-bulking by a gynecologic oncologist, followed by multiple rounds of chemotherapy with paclitaxel and carboplatin. Disease burden is monitored by measuring serum levels of the biomarker CA125. This aggressive approach typically provides a temporary remission, followed by the emergence of treatment-resistant ovarian cancer despite use of additional targeted therapeutics (e.g. PARPi, doxil, bevacizumab, etc.), and thus most patients succumb to their disease. Although a chimeric IgG1 directed against Folate Receptor α and cleavably-coupled to DM4 microtubule-disrupting agent was recently licensed for treating Folate Receptor α positive, platinum-resistant epithelial ovarian cancer, there remains an urgent need for new targeted treatment approaches to treat or prevent ovarian cancer.

MUC16 is a massive and highly glycosylated cell surface protein present in large amounts on the surface of almost all ovarian cancer cells. MUC16 sheds a piece called CA125 into serum and other body fluids. While measurement of CA125 in serum is a blood test used to monitor treatment of ovarian cancer, it is not sufficiently predictive for use in screening [2]. Since MUC16 (and its fragment CA125) contributes to the evasion of the body’s immune defense against the cancer cells, as well as directly promoting cancer cell growth and spread [3], there have been major efforts to target MUC16 with antibodies (Oregovomab) and/or vaccines (Abagovomab) to better treat ovarian cancer [3].

Oregovomab is a modified version of the mouse IgG1κ monoclonal antibody MAb B43.13 that binds to CA125. It was initially developed as a tumor imaging agent and administration of technetium-99 labeled B43.13 was associated with an unexpectedly better survival for ovarian cancer patients [4–6]. In a phase II study of Oregovomab monotherapy in patients with recurrent disease, robust immune responses to the monoclonal antibody were detected in 58%, and disease stabilization occurred in 23% of ovarian cancer patients. Oregovomab treatment was also well tolerated in combination with salvage chemotherapy. In the setting of maintenance therapy in patients showing complete clinical response, Oregovomab treated patients (n = 73) exhibited a median survival of 57.5 months versus 48.6 months for the placebo control patients (n = 72), but this did not reach significance (hazard ratio 0.72, 95% CI 0.41–1.25) [7, 8]. Nevertheless, in a phase III study no differences in the median time to relapse were observed: 10.3 months (95% CI, 9.7–13.0) for oregovomab versus 12.9 months (95% CI, 10.1–17.4) for the placebo arm (*p* = 0.29) [8]. A phase III trial (FLORA-5, NCT04498117) evaluating a concomitant schedule of oregovomab with chemotherapy in new diagnosed OC patients, is ongoing. Taken together, these observations suggest that passive immunization against CA125 may not be sufficient, and that active immunization may be needed.

Abagovomab is a related monoclonal antibody that was developed as a vaccine to trigger an anti-idiotype CA125-specific antibody response (Ab3) [9–11]. Abagovomab failed in a pivotal international phase III study in patients with CA125+ advanced ovarian cancer in remission following surgery and standard chemotherapy to impact progression-free and overall survival [10]. This suggests that the epitope which resides in the shed CA125 may be the wrong target within MUC16 [3], and that the anti-idiotype approach only weakly induces CA125 antibodies, i.e. it is a poorly immunogenic vaccine [11, 12].

Over-expression of the C-terminal 114 amino acids of MUC16 has been shown to promote transformation and tumor invasion in mouse models [13–15]. These 114 amino acids include 12 extracellular residues (PLTGNSDLPFWA), the single transmembrane domain and a short intracellular domain [16]. The oncogenic changes observed were dependent on the membrane-proximal extracellular MUC16 sequences [17], and the glycosylation sites [18]. Targeting this region with antibody and/or vaccines may prevent this tumor-promoting function by blocking signaling and/or triggering endocytosis and degradation.

The human MUC16 epitope targeted by the vaccines herein (GYSPNRNEPLTGNSDLPFWA) includes these key membrane proximal twelve amino acids based on the hypothesis that the vaccination-induced antibody should not target the bulk of MUC16 that is shed, but rather this stub that remains on the tumor cell surface after release of CA125 [16]. Targeting of this stub of MUC16 with antibody may be important because the C-terminal tail of MUC16 activates signaling events after cleavage that promote oncogenesis [17]. Further, coating the surface of MUC16 + cancer cells with a high density of IgG may opsonize and trigger antibody-dependent cellular cytotoxicity (ADCC), complement-dependent cytotoxicity (CDC) and other Fc-mediated antitumor effects [19].

Many viral structural proteins have the intrinsic ability to self-assemble into VLPs that are non-infectious but structurally similar to virus [20]. VLPs make excellent vaccines because the regularity of their capsid structure presents viral epitopes as dense, highly repetitive arrays that strongly stimulate B cells and induce high titer antibody responses. VLPs can be used as the basis for vaccines targeting the virus from which they were derived. Human Papillomavirus (HPV) VLPs are one licensed example produced by recombinant expression of the L1 major capsid protein. HPV VLPs are highly immunogenic and durably protective in patients with even a single dose formulated on alum adjuvant. Importantly, these VLPs also can be used as platforms to display heterologous epitope in a multivalent format by genetic insertion into a surface loop of L1 [21] or chemical coupling to their surface. These recombinant VLPs induce strong antibody responses at low doses and often without requiring co-administration of adjuvant [22]. Strikingly, even self-antigens, which are normally subject to the mechanisms of B cell tolerance, are highly immunogenic when displayed at high density on the surface of VLPs [23]. Display of epitopes on L1 VLP has been exploited to develop vaccines against self-molecules that effectively prevent or reduce the severity of disease in animal models of Alzheimer’s Disease, rheumatoid arthritis, and HIV infection [24, 25]. For example, vaccination with TNFα displayed on L1 VLPs induced high-titer (> 10^4^) anti-TNFα antibody that prevented collagen-induced arthritis in a mouse model [26]. Clinical trials of VLP-based vaccines targeting self-antigens that are involved in Alzheimer’s disease, rheumatoid arthritis, and hypertension have shown that this approach is effective in inducing self-antibody responses in humans [20].

Here we compare HPV16 VLP display of a 20aa MUC16 epitope by genetic insertion in an immunodominant surface loop of L1, or by chemically coupling it to L1 cysteine residues on the outside of the capsids to produce MUC16-VLP vaccines. The regular and close-packed display of MUC16 peptide on the surface of HPV16 VLPs is intended to safely break tolerance and thus generate a robust MUC16-antibody response targeting ovarian cancer cells.

## Results

### Genetic insertion of MUC16 juxta-membrane epitope into HPV16 L1 VLP

**Fig. 1:**
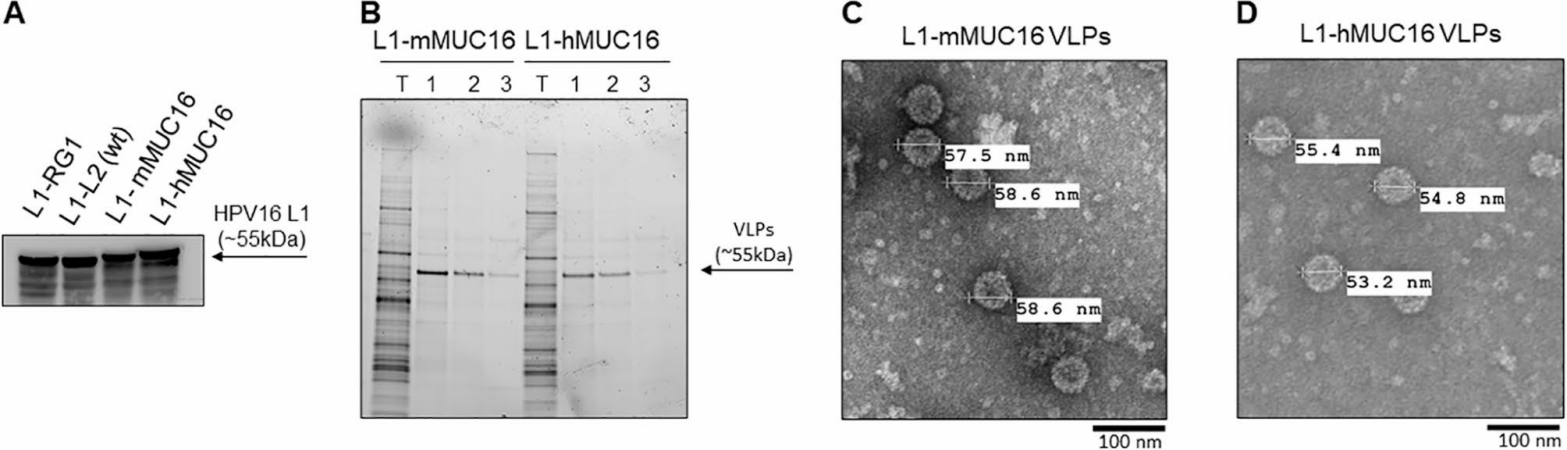
Assembly of VLP displaying human or mouse MUC16 20mer epitopes. **(A)**. HEK293 cells were transfected with expression constructs for wild type (wt) HPV16 L1 and L2 (L1-L2) or HPV16 L1 with 20 aa inserts of the RG1 epitope (L1-RG1), mMUC16 (L1-mMUC16) or hMUC16 (L1-hMUC16). Three days later, cell lysates were harvested and subjected to Western blot analysis with L1-specific monoclonal antibody, CamVir-1. Lysates of HEK293 cells transduced with L1-mMUC16 or L1-hMUC16 expression vectors were fractionated on an Optiprep step gradient, and VLP-containing fractions analyzed by SDS-PAGE and Coomassie staining. **C** and **D)**. Transmission electron microscopy of fraction 1 from **B** for L1-mMUC16 (**C**) or L1-hMUC16 (**D**)

A DNA vaccine was recently licensed for the prevention of COVID19 [27], and given this success we first attempted to generate DNA vaccines based upon the pcDNA3.1(+) expression vector. A codon-optimized HPV16 L1 gene encoding full length HPV16 L1 with either the murine MUC16 (mMUC16) or human MUC16 (hMUC16) extracellular membrane-adjacent 20 aa peptide inserted into the capsid surface D-E loop was synthesized and inserted into the pcDNA3.1(+) vector. Expression of the L1-mMUC16 and L1-hMUC16 inserts was determined by transfection of HEK 293 cells with each plasmid or a control plasmid expressing HPV16 L1 with an epitope of HPV16 L2 17–36 (recognized by monoclonal antibody RG1) inserted in to the D-E loop of HPV16 L1 [28]. Western blot analysis using an HPV16 L1-specific monoclonal antibody (Camvir-1) revealed similar levels of expression of the HPV16 L1 fusions with D-E loop insertions of the mMUC16, hMUC16 and the control RG1 epitopes in detergent lysates of 293 cells at 3 days post transfection as compared to wild type (wt) HPV16 L1 co-expressed with HPV16 L2 (Fig. 1A). The apparent molecular weight of the mMUC16 and hMUC16 fusions were slightly greater than for the wild type L1, as expected given their extra 20aa insert.

To examine whether the HPV16 L1 fused to either mMUC16 or hMUC16 could produce VLPs when over-expressed in 293 cells, the lysates were subjected to OptiPrep density gradient centrifugation. The gradients were fractionated and fractions with the expected buoyant density examined by SDS-PAGE and Coomassie-staining (Fig. 1B). The presence of bands corresponding to L1-mMUC16 and L1-hMUC16 was evident in fractions at the bottom of the gradients, wherein HPV16 L1-VLPs are typically found. Examination of these fractions by transmission electron microscopy (TEM) revealed the presence of ~ 50–60 nm diameter VLPs produced by expression of L1-mMUC16 and L1-hMUC16. The L1-mMUC16 and L1-hMUC16 VLPs had the typical morphology, including regular assemblies of donut-shaped capsomers (Fig. 1C,D). Unfortunately, the efficiency of VLP assembly was dramatically reduced by the insertion of either MUC16 epitope, where capsomers predominate. Therefore, we elected to continue with DNA rather than protein-based vaccination with VLPs.

### Vaccination with DNA expressing HPV16 L1 VLP displaying MUC16 epitope

**Fig. 2:**
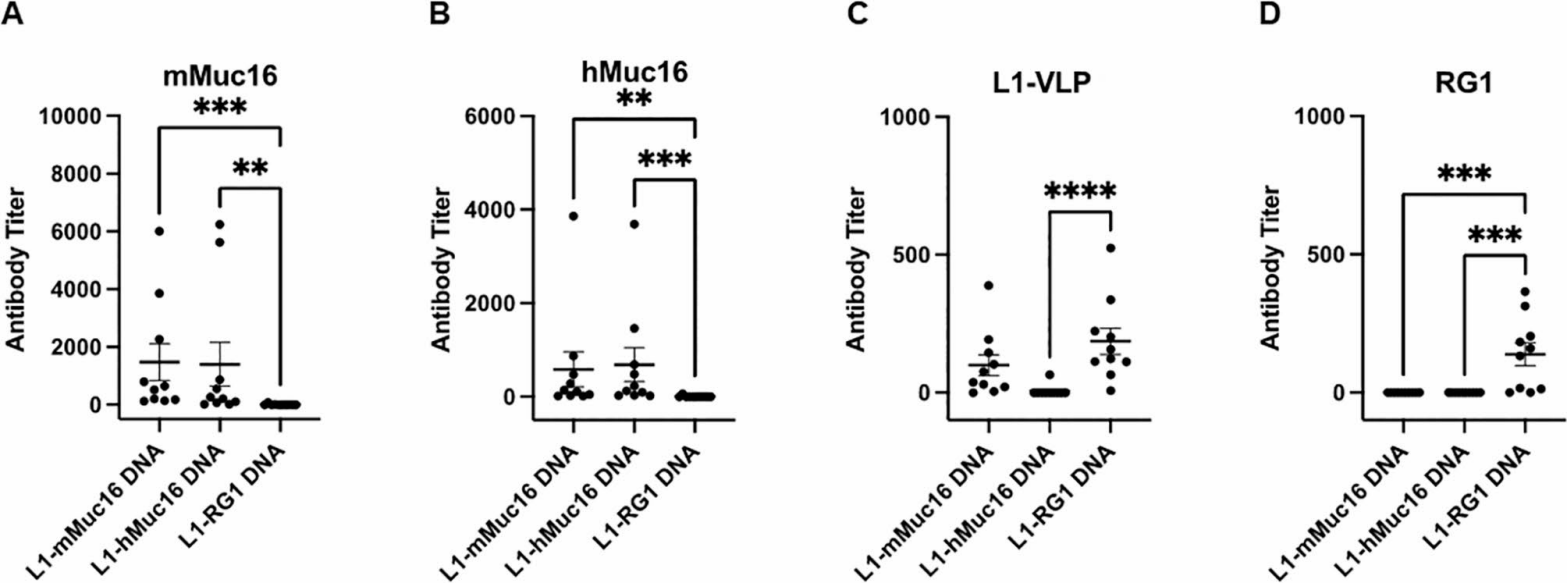
ELISA of antibody response to vaccination with DNA vectors expressing L1 with 20aa inserts of the RG1 epitope, mMUC16 or hMUC16. Female mice (n = 10/group) were vaccinated 3 times i.m. followed by electroporation with 30 µg DNA in PBS, and serum was harvested 2 weeks later (**A-D**). Sera were analyzed by ELISA against mMUC16 (**A**), hMUC16 (**B**) or RG1 (**D**) 20mer N-terminally biotinylated peptide on streptavidin coated plates, or HPV16 L1-VLP coated plates (**C**)

Female C57BL6 mice (n = 10/group) were vaccinated three times at 19 day intervals with each DNA by intra-muscular injection and electroporation. Serum was collected 2 weeks later for analysis of the antibody responses by ELISA as our prior studies suggest the peak antibody response occurs at this time (Fig. 2). Sera were diluted 2-fold from 1:50, and a titer of < 50 was recorded as 0. The sera from mice vaccinated with DNA expressing L1 fused to mMUC16 and hMUC16 elicited similar antibody titers specific for either mMUC16 or hMUC16 peptides (Fig. 2A,B). The antibody response reactive to mMUC16 peptide suggests that B cell tolerance was broken. The similarity in the antibody responses to hMUC16 and mMUC16 is consistent with their minimal differences in sequence. The control DNA vaccine expressing HPV16 L1-RG1 elicited no detectable antibody that bound to either mMUC16 or hMUC16 peptides, as expected. Conversely, this DNA vaccine elicited a robust response to the RG1 peptide, whereas the DNA vaccines expressing L1 fused to mMUC16 and hMUC16 did not (Fig. 2D). The DNA vaccines expressing HPV16 L1-mMUC16 and HPV16 L1-RG1 induced a significant but low antibody titer against purified HPV16 L1-VLP. Surprisingly the L1-hMUC16 DNA construct did not produce a detectable response (Fig. 2C). All of these mice were maintained for 5 months post-vaccination, and remained apparently healthy.

### Chemical coupling of MUC16 epitope to the surface of HPV16-derived VLP

Given the global use of HPV vaccines, we examined both mixing of MUC16 peptides with pre-formed, purified HPV16 L1-VLP and chemical coupling of MUC16 peptides with N-terminal malemide modification to the sulfhydryl moiety of cysteine residues on the surface of HPV16 L1-VLPs. Prior work suggests that the density of surface display of self-epitopes is important in breaking tolerance and inducing high titer and durable antibody responses [26, 29, 30]. Ishii et al. showed that HPV16 L1 C146, C225, and C229 have free thiol on the surface of VLP [31]. Since the RG1 epitope contains two cysteine residues, we also sought to chemically couple MUC16 peptides with N-terminal malemide modification to HPV16 L1-RG1-VLP because of a potentially higher display of surface cysteine residues as compared to HPV16 L1-VLP (5 instead of 3).

**Fig. 3:**
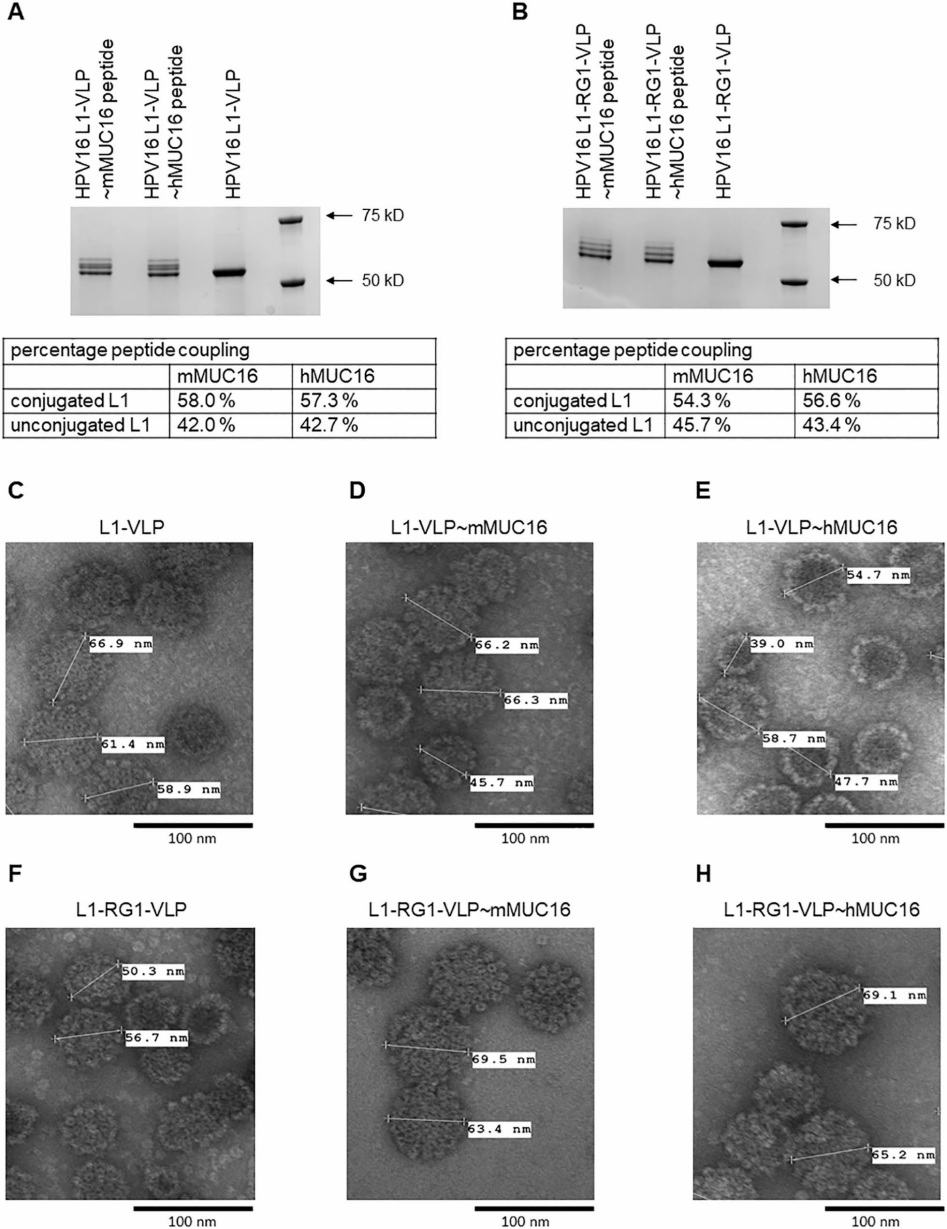
Malemide coupling chemistry to covalent link MUC16 peptide to surface sulfhydryl on L1 VLP. Purified HPV16 L1-VLP (**A**) or L1-RG1-VLP (**B**) were reduced and coupled with malemide-linked mMUC16 or hMUC16 peptide. Samples and molecular weight standards (right of panel) were separated by SDS-PAGE and the gels stained with Coomassie (**A, B**), or the samples were viewed by transmission electron microscopy (**C-H**).

Prior to conjugation, both the HPV16 L1-VLP and HPV16 L1-RG1-VLP preparations were reduced using TCEP (tris 2-carboxyethyl phosphine) in a 10:1 (TCEP:VLP) molar ratio. After incubation in reaction buffer at room temperature for 1 h, N-terminal malemide-modified mMUC16 or hMUC16 peptide was added in a 5:1 (peptide:HPV16 L1-VLP) or 10:1 (peptide:L1-RG1-VLP) molar ratio. The conjugated VLP was then dialyzed into storage buffer to remove reactants and analyzed by SDS-PAGE. The conjugated L1 is seen as a ladder of bands with apparent molecular weights above the 55 kDa band of unconjugated L1 capsid protein; the band immediately above the unconjugated band was considered to have one molecule of peptide attached per molecule of L1, and the second conjugated band would have 2 peptides per L1, and so on. The percentage of peptide coupling was determined using ImageLab software (Biorad Laboratories), where the sum of density of the upper bands corresponds to L1 conjugated with peptide(s) as compared to the density at 55 kDa which represents unmodified HPV16 L1. For the mMUC16 and hMUC16 peptides, 58% and 57.3% of the L1 in HPV16 L1-VLP was conjugated (Fig. 3A), whereas in the L1-RG1-VLP 54.3% and 56.6% of the L1-RG1 chimeric protein was conjugated (Fig. 3B). Furthermore, the banding pattern by SDS-PAGE was similar in all four reactions, suggesting mostly 1 or 2 peptides coupled per capsid antigen, and rarely 3. The structural integrity of the conjugated VLP was visualized using TEM. TEM of the coupled VLPs showed normal morphology and typical diameter of 50–60 nm (Fig. 3C-F). This suggests that the coupling process did not compromise the particle structure. However, some larger particle diameters approaching 100 nm were evident, suggesting possible fragility or artifact of the staining since this was also seen pre-conjugation.

### Vaccination of mice with VLP chemically coated with MUC16 peptide

**Fig. 4:**
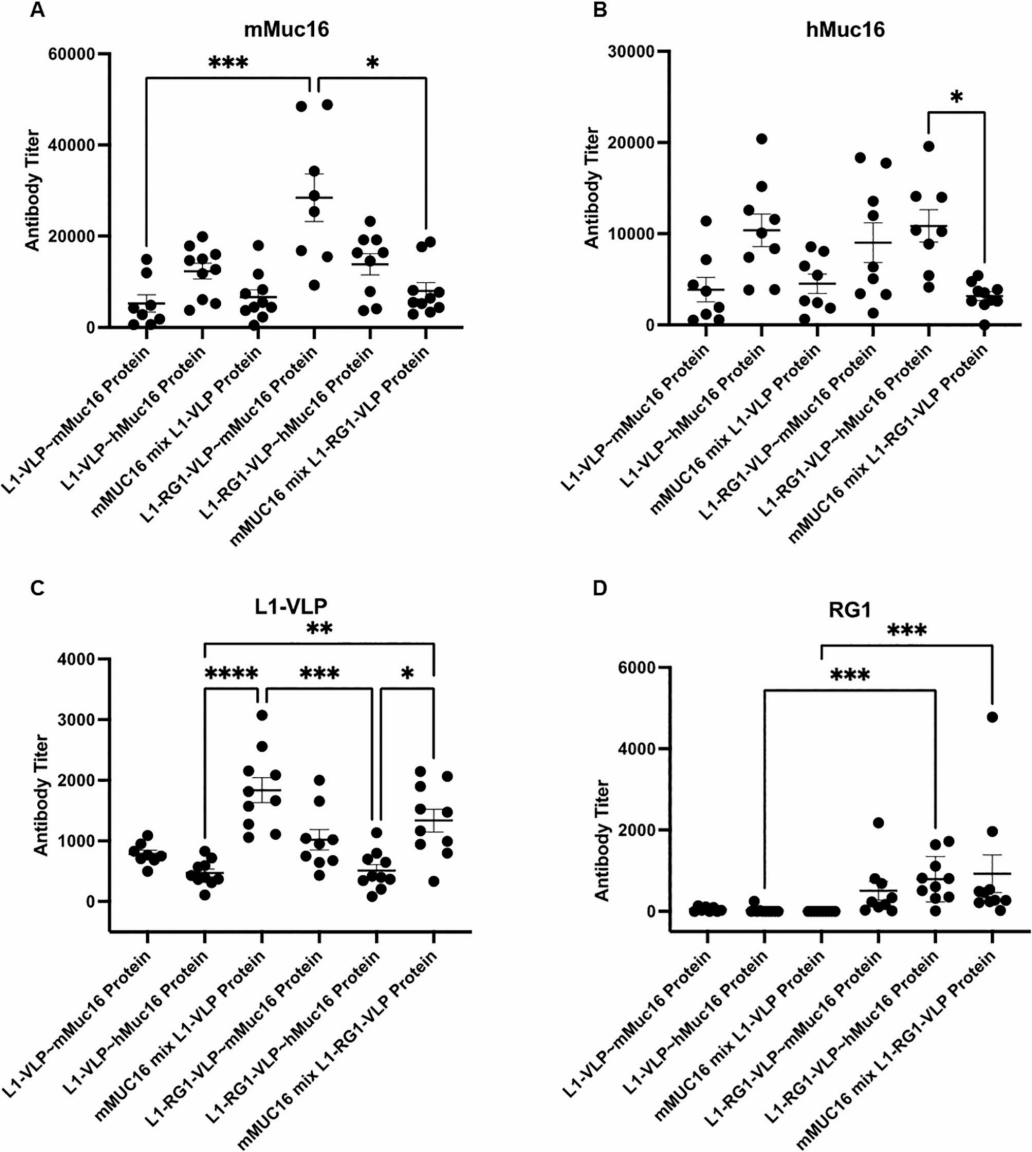
ELISA of antibody response to vaccination with VLP either mixed with or coupled to 20aa mMUC16 or hMUC16 peptides. Female mice (n = 10/group) were vaccinated 3 times i.m. with alum-formulated L1 VLP or L1-RG1-VLP VLP either mixed (mix) with or coupled (~) to 20aa mMUC16 or hMUC16 peptides, and serum was harvested 2 weeks later (**A-D**). Sera were analyzed by ELISA against mMUC16 (**A**), hMUC16 (**B**) or RG1 (**D**) 20mer N-terminally biotinylated peptide on streptavidin coated plates, or HPV16 L1 VLP coated plates (**C**)

Eight week-old C57BL/6 female mice (10 per group) were vaccinated via intramuscular injection with the following protein preparations formulated on aluminum hydroxide (alum): HPV16 L1-VLP conjugated with either mMUC16 (L1-VLP ~ mMUC16) or hMUC16 peptide (L1-VLP ~ hMUC16), or HPV16 L1-RG1-VLP conjugated with either mMUC16 (L1-RG1-VLP ~ mMUC16) or hMUC16 peptide (L1-RG1-VLP ~ hMUC16). To determine the influence of coupling the peptides to VLPs upon their immunogenicity, groups of mice were also injected with either HPV16 L1-VLP mixed with equivalent amount of loose mMUC16 peptide (mMUC16 mix L1-VLP) as was conjugated to the VLP in the other preparations, or L1-RG1-VLP mixed with the same amount of loose mMUC16 peptide (mMUC16 mix L1-RG1-VLP). Since there were an average of ~ 1 peptide coupled to each L1 as seen in SDS-PAGE (Fig. 3A,B), the calculation of the amount of loose peptide added was based on one molecule of peptide conjugating to one molecule of L1. Each mouse received 10 µg of VLP (conjugated or with loose peptide), 50 µg of Alhydrogel® (vac-alu-250, Brenntag Biosector) in 100 µL PBS for three times 19 days apart. As negative controls, 5 mice were not vaccinated. Two weeks after the last injection, blood was collected and serum was separated and stored at -20^o^C until analysis by ELISA (Fig. 4).

The sera from mice vaccinated with L1-RG1-VLP coupled to mMUC16 peptide demonstrated significantly higher antibody titers specific for mMUC16 peptide as compared to L1-VLP conjugated with mMUC16 peptides (Fig. 4A). Likewise, mice vaccinated with L1-RG1-VLP coupled to hMUC16 exhibited a higher antibody response specific for hMUC16 peptide than mice vaccinated with L1-VLP coupled to hMUC16 (Fig. 4B), although it did not quite reach significance. These findings suggest that an increased density of coupling is associated with a higher antibody response.

A surprise is the robust mMUC16 and hMUC16-specific antibody response in sera of mice vaccinated with VLP mixed with mMUC16 peptide, suggesting that the L1 contributes powerful T help and that this combination is able to break tolerance. Indeed it is similar in titer to the MUC16-specific antibody response in sera of mice vaccinated with L1-VLP coupled to MUC16 peptide (Fig. 4A,B). Nevertheless, both the mMUC16 and hMUC16-specific antibody responses in sera of mice vaccinated with L1-RG1-VLP coupled to mMUC16 peptide is significantly stronger than when mixed with these particles.

It is noteworthy that vaccination with VLPs coupled to hMUC16 induced antibodies that cross-reacted with mMUC16 peptide (Fig. 4A). Likewise, vaccination with VLPs coupled to hMUC16 induced antibodies that reacted with mMUC16 peptide (Fig. 4B), although the reactivity to hMUC16 trended slightly higher than in sera from mice vaccinated with VLPs coupled to mMUC16. These finding are consistent with the similarity in sequence of these epitopes of hMUC16 and mMUC16.

Vaccination with all particle preparations generated a robust antibody response to HPV16 L1-VLP when measured by ELISA using sera collected 2 weeks post the third immunization (Fig. 4C). The antibody response against HPV16 L1-VLP that is elicited upon vaccinating with L1-VLP or L1-RG1-VLP was not significantly different, as expected since the RG1 peptide is only a minor constituent of the particle. This is consistent with prior data [32]. The L1-VLP reactive antibody responses produced by vaccination with the preparations in which the MUC16 peptides were coupled to the VLP were significantly lower than for the preparations where the particles were simply mixed with the peptides, regardless of whether the peptides were derived from human or mouse MUC16 sequences. This implies that coating of the VLP with the peptides may mask B cell recognition of the immunodominant L1 surface epitopes, resulting in lower antibody responses (Fig. 4C).

As expected, responses to the RG1 peptide were only seen in animals vaccinated with L1-RG1-VLP (Fig. 4D). The RG1 peptide-specific response was not significantly different in the sera of mice vaccinated with L1-RG1-VLP mixed with mMUC16 peptide versus coupled to mMUC16 or hMUC16 peptide. This suggests that the two cysteines in the RG1 epitope displayed on L1-RG1-VLP were not as susceptible to coupling with peptide, perhaps because they preferentially link to one another. Indeed the degree of peptide coupling to HPV16 L1 VLP and L1-RG1-VLP appeared quite similar based upon the SDS-PAGE analysis (Fig. 3A,B). Presumably the coupling predominated at the surface cysteines (C146, C225 and C229) of HPV16 L1 previously identified as being available of the surface of VLP for modification with thiol reagents [31].

Analysis of the isotypes of the serum antibodies to hMUC16 peptide revealed a balanced IgG response at 2 weeks post the third intramuscular immunization. Overall, higher responses to IgG1 and IgG2b than IgG2a or IgG3 were observed for the protein-based vaccination regimens. By contrast the IgG2b isotype dominated in the response to DNA-based vaccination, followed by IgG2a, but minimal IgG1 or IgG3 hMUC16-specific antibody was detected. The κ light chain response was higher than λ; no significant IgA response and only a weak IgM response was seen regardless of MUC16 vaccine type.

### Serologic reactivity with human MUC16 in ovarian cancer cells

**Fig. 5:**
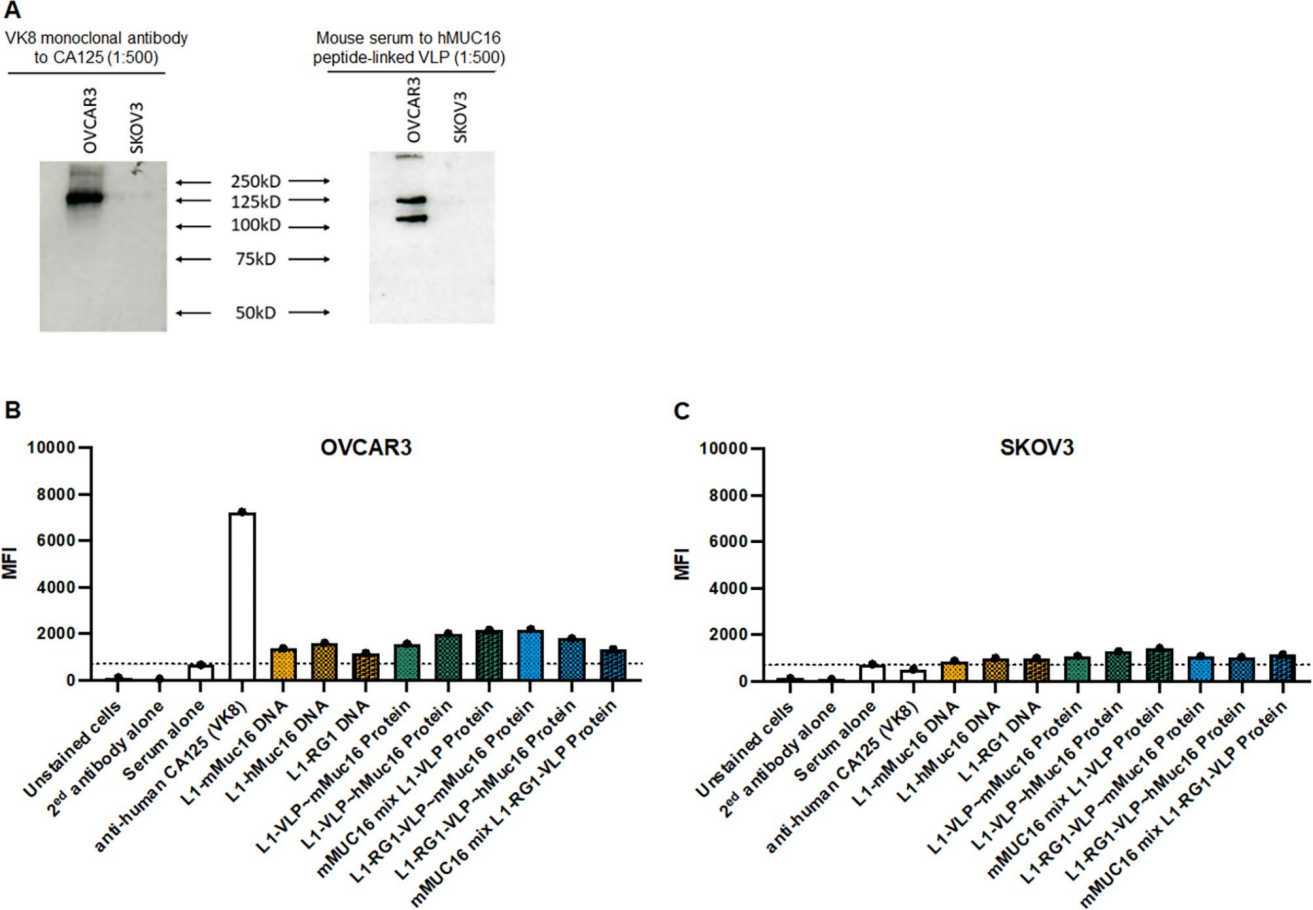
Binding of MUC16-specific antibody to CA125 + ovarian cancer cell surface and lysates. **A)** Monoclonal antibody VK8 to CA125 and serum of a mouse vaccinated with hMUC16 peptide coupled to L1-RG1-VLP was used in an immunoblot to probe cell lysate of OVCAR3 (positive control) or SKOV3 (negative control). **B and C)**. Monoclonal antibody VK8 or mouse antisera described in Figs. 2 and 4 were bound with the surface of intact OVCAR3 (**B**) or SKOV3 cells (**C**). After washing, bound mouse antibody was detected using PE-labeled anti-mouse IgG and flow cytometry. Mean fluorescent intensity (MFI) was determined

The human ovarian cancer cell line OVCAR3 is reported to express high levels of MUC16 whereas SKOV3 does not. To confirm this, lysates from each cell line were probed by Western blot with a mouse monoclonal antibody, VK-8, to the CA125 fragment of MUC16. Reactivity was evident in the OVCAR3 lysate, but none was detected in the SKOV3 lysate (Fig. 5A). Serum of a mouse vaccinated against hMUC16-coupled L1-RG1VLP was also tested by Western blot for reactivity against these cell lines. Reactivity against ~ 115 and ~ 125 KDa was apparent solely in the OVCAR3 lysate (Fig. 5A), likely corresponding to the C-terminus of MUC16. However, we did not see the previously reported 17 kDa band corresponding to the C-terminal stub of MUC16 previously described by Aithal et al. using monoclonal antibody 5E6 [33]. It is possible that our MUC16 peptide-specific antiserum fails to detect this 17 kDa band because the proteolytic cleavage that generates this fragment occurs in the middle of the MUC16 peptide used in our immunizations (i.e. the epitope is disrupted in making the 17 kDa MUC16 fragment). Alternatively, the difference may reflect our use of M-PER to extract membrane proteins from OVCAR3 cells as compared to the much stronger extraction buffer RIPA in the prior study.

Flow cytometry of intact OVCAR3 and SKOV3 cells was used to determine serum antibody mean fluorescent intensity (MFI) of reactivity with their cell surface. The positive control VK-8 mouse monoclonal antibody strongly reacted with OVCAR3 cells, whereas the signal on SKOV3 cells was below that of unvaccinated mouse serum. Consistent with the hMUC16 peptide ELISA data (Fig. 2B), the reactivity to OVCAR3 cells of the pooled sera of mice vaccinated with L1-mMUC16 or L1-hMUC16 expressing DNA was greater than for L1-RG1 DNA vaccinated mice (Fig. 5B). Conversely, in SKOV3 cells that do not express MUC16, the MFI of pooled sera of mice vaccinated with L1-mMUC16 and L1-hMUC16 expressing DNA was similar to, or less than that for negative control sera from L1-RG1 vaccinated mice (Fig. 5C).

The serum reactivity to hMUC16 peptide of mice that received protein-based vaccination with alum adjuvant was approximately 10-fold higher in ELISA titer (Fig. 4B) when compared to the sera of mice vaccinated with L1-mMUC16 or L1-hMUC16 expressing DNA (Fig. 2B). Likewise, greater MFI of reactivity to OVCAR3 was seen with these pooled sera by flow cytometry, as compared the sera of mice vaccinated with L1-mMUC16 and L1-hMUC16 expressing DNA (Fig. 4B). Conversely, the MFI of reactivity to SKOV3 seen with these pooled sera by flow cytometry, was similar to the negative control sera of mice vaccinated with L1-RG1 expressing DNA or serum of naïve mice. This suggests that the observed reactivity to OVCAR3 was specific to MUC16. However, it is weak as compared to the VK-8 monoclonal antibody. This might be expected since these polyclonal antisera target a single 20aa peptide per MUC16 protein molecule, whereas VK-8 binding to MUC16’s highly (28x) repeated CA125 epitope and it is monoclonal in specificity [3].

## Discussion

Genetic insertion of the membrane proximal 20 aa of the ectodomain of MUC16 into an immunodominant surface loop of HPV16 L1 yielded VLP upon its overexpression in mammalian cells, but the efficiency of assembly was greatly reduced compared to wild type L1. This led us to seek alternate means of using VLP to trigger MUC16-specific antibody responses. Intramuscular vaccination three times with the DNA expressing L1-mMUC16 followed by in vivo electroporation elicited mMUC16 peptide-specific antibody responses. This suggesting the ability to break B-cell tolerance against this sequence despite the wide expression of MUC16 in adult mouse tissues. Indeed, Xing et al. showed MUC16 expression on the lining epithelia of the trachea, the secretory glands in the oral cavity, the surface of the olfactory epithelia, and on mesothelial cells lining body cavities (i.e., pleural, peritoneal, and pelvic cavities), and male and female reproductive organs [34]. In addition, MUC16 protein is expressed on the surface epithelia of the cochlear duct and chief cells of the stomach, suggesting multiple physiologic roles for MUC16 and thus the potential for autoimmune disease upon induction of MUC16-specific antibodies [34]. Nevertheless, vaccination against mMUC16 was tolerated, perhaps reflecting protection by MUC16’s glycocalyx from antibody binding adjacent to the membrane. By contrast, proteases in the tumor microenvironment cleave away large portions of the MUC16 ectodomain, potentially uniquely exposing the 20aa membrane adjacent epitope for antibody binding in the context of cancer.

Patients can spontaneously generate CA125-specific autoantibody responses [35, 36] which have been associated with extended survival [37]. Likewise, passive or active immunization with monoclonal antibodies against CA125 was well tolerated, although not associated with a proven survival benefit [8, 12].

Interestingly, there was cross-reactivity to human MUC16 peptide by antibody responses upon mMUC16 peptide vaccination and vice versa. This is consistent with significant sequence conservation between these two sequences (Mouse sequence: **GYSQNRDDDVMKNSGLPFWA**; Human sequence: **GYS**P**NR**NEPLTG**NS**D**LPFWA**). Indeed, vaccinating with xenogenic sequences can also help overcome tolerance to self-antigens [38].

Intramuscular vaccination with DNA followed by electroporation has been studied extensively in patients targeted a variety of pathogen-derived [39–42] and self-antigens [43] and has been well tolerated. Nevertheless, it is complicated by the need for the electroporation device and the electrical stimulation can be more painful [44] than conventional protein-based vaccination. A more robust antibody response to MUC16 might be achieved by combining L1-VLP (purified from yeast or Sf9 insect cells, for example) that display MUC16 epitopes with adjuvants like alum and monophosphoryl lipid A, as utilized in the licensed Gardasil and Cervarix vaccines. The ability to produce papillomavirus virus-like particles (e.g. Gardasil, Cervarix) and their safety in millions of children and young adults and robust immunogenicity is well documented. Indeed, single dose vaccination elicits durable immunity over at least a decade [45, 46]. The IgG isotypes in the hMUC16-specific antibody response was balanced for protein vaccination, but dominated by IgG2b for DNA-based vaccination. The isotype balance may have implications for ADCC, CDC, phagocytosis and other Fc-mediated effects, and can be influenced by the type of adjuvant utilized. A weakness of this study was a failure to assess immunogenicity with fewer than 3 doses.

The L1-hMUC16 DNA vaccination did induce a weak antibody response to the hMUC16 epitope, but no significant antibody response to wild type HPV16 L1-VLPs. This suggests that the vaccination was technically successful, but the antibodies induced failed to recognize the HPV16 capsid. The failure of the antibodies to recognize native HPV16 L1-VLPs implies that the insertion of the hMUC16 sequence into HPV16 L1 negatively impacts the assembly efficiency of VLPs and/or significantly alter their structure. Indeed, we observe that insertion of the MUC16 epitope does profoundly reduce the efficiency of VLP assembly, although the morphology of the few particles by transmission electron microscopy is indistinguishable from native HPV16 L1 VLP. Furthermore, the VLPs produced by plasmid expression are targeted to the nucleus (i.e. they are not released), and the DNA vaccination is given without an adjuvant, factors which may also account for their low immunogenicity. These issues are addressed by conjugating the peptides to the outside of purified VLPs and then vaccinating with them in the presence of adjuvant.

Since genetic insertion of MUC16 epitope into the selected site of L1 compromised VLP assembly, we resorted to chemical coupling of synthetic MUC16 peptide to free cysteines accessible on the surface of VLP using malemide chemistry. The chemical coupling of the MUC16 epitopes to VLP was challenging because disulphide bonding is important for stabilizing VLP assembly. Nevertheless, a protocol was developed that conferred coupling to about half of each of the 360 L1 per VLP. Based upon the banding pattern in the SDS-PAGE analysis, typically one or two peptides and up to 3 were coupled per L1, suggesting a total of ~ 300 peptides/particle. Although not specifically identified herein, it is likely that the sulphydryls of the 3 known surface cysteine on HPV16 L1 (C146, C225 and C229) are subject to modification with the maleimide-linked MUC16 peptides [31]. The extent of labeling with the maleimide-linked MUC16 peptides was similar for the L1-VLP and L1-RG1-VLP, although the bandings pattern (Fig. 3A,B) differed implying that the distribution of the coupling may differ subtly. That the extent of labeling with the maleimide-linked MUC16 peptides was similar for L1-VLP and L1-RG1-VLP, despite the extra pair of surface cysteine residues within the RG1 epitope of the latter, implies they likely disulphide bond to one another instead. If this is the case, it negates the rationale of utilizing the L1-RG1-VLP over L1 VLP as a coupling substrate for maximum density of peptide display. Indeed, while the mMUC16-specific antibody response trended higher in sera of mice vaccinated with L1-RG1-VLP versus L1 VLP coupled to mMUC16 (Fig. 4A), the hMUC16-specific antibody response was similar in sera of mice vaccinated with L1-RG1-VLP versus L1 VLP coupled to hMUC16 (Fig. 4B).

A consideration for using HPV16 L1 as a carrier for the MUC16 peptide is the potential impact of prior vaccination of patients with Gardasil [9] or Cervarix upon the MUC16-specific antibody response. If prior HPV vaccination does compromise the MUC16-specific antibody response, then an alternative would be to use L1 derived from an HPV genotype that is not targeted by any licensed vaccine, or perhaps even an animal papillomavirus, as a carrier. Indeed, the genetic insertion and surface labeling approaches have both been successfully used with bovine papillomavirus type 1 L1-VLP [47, 48]. Interestingly, chemically coating the VLPs with MUC16 peptide substantially suppresses the HPV16 L1 VLP-specific antibody response (as compared to simply mixing the VLP with peptide). This may reduce the concern of pre-existing HPV vaccination if most L1-derived VLP surface epitopes are masked by MUC16 peptide.

While coupling of mMUC16 peptide to L1-RG1-VLP increased its immunogenicity in mice, remarkably robust peptide-specific antibody responses were induced simply by mixing mMUC16 peptide with either L1 VLPs or L1-RG1-VLPs prior to vaccination. This suggests that both the L1 VLPs or L1-RG1-VLPs can provide strong T cell help. Further, it is a surprising finding for a self-antigen, but might be explained if this epitope is normally masked by MUC16’s massive glycocalyx. These mMUC16-specific antibody responses to vaccination with mMUC16 peptide mixed with particles were significantly weaker than for the L1-RG1-VLP-coupled, but not L1 VLP-coupled peptide preparations (Fig. 4A). Nevertheless, simply mixing hMUC16 peptide with a licensed HPV vaccine might prove a simpler alternative to test this approach clinically.

Monoclonal antibodies are being developed to target an extracellular MUC16 epitope adjacent to the membrane, e.g. 5E6 which binds residues NFTLDRSSVLVDGYSPN [33, 49], partially overlapping the DGYSPNRNEPLTGNSDLPFWA region targeted herein. Administration of MUC16-specific monoclonal antibodies has some inherent advantages, notably that treatment can be stopped in the event of toxicities, and the antibodies can be derivatized with cytotoxic or imaging agents [49–55] or rendered bi-specific [56], or switched for isotype or modified for glycosylation or sequence. However, they must generally be repeatedly administered in high doses at significant cost. Conversely, vaccination to target MUC16 would likely require few administrations at low doses. However, a vaccine-based approach requires that immune tolerance to MUC16 is broken with associated risks of autoimmune disease. Importantly, in contrast to administration of a MUC16-specific monoclonal antibody, vaccine-induced MUC16 antibodies cannot be withdrawn in the event of toxicity.

## Conclusions

Our study provides support for further optimization and testing of MUC16-targeted VLP vaccines based upon papillomavirus L1, including the safety of the approach in animals and its ability to prevent and limit the growth of mouse and human models of ovarian cancer. Based upon our data herein, vaccination against the juxta-membrane region of the MUC16 ectodomain is likely to be well tolerated and induce antibody responses targeting CA125 + ovarian cancer cells.

## Methods

### Ethics approval

All procedures were performed according to approved protocols by the Johns Hopkins Medical Institutions Animal Care and Use Committee and the National Institutes of Health. All mice were housed and handled in the animal facility of the Johns Hopkins Medical Institution under specific-pathogen-free conditions.

### Mice

Six-week-old female C57BL/6 mice (strain 027) were purchased from Charles River Laboratories). All mice were maintained at the Johns Hopkins University School of Medicine (Baltimore, MD) animal facility under specific pathogen-free conditions.

### Generation of, and vaccination with DNA encoding HPV16 L1 with MUC16 or RG1 epitope insertion

A codon-optimized gene encoding HPV16 major capsid protein L1 isolate 114B [57] containing an insertion of the extracellular membrane-adjacent region of mouse MUC16 (GYSQNRDDDVAKNSGLPFWA), or human MUC16 epitope (GYSPNRNEPLTGNSDLPFWA) or the HPV16 L2 17–36 (QLYKTCKQAGTCPPDIIPKV) epitope of monoclonal antibody RG1 [58], between amino acids 136 and 137 was directly synthesized (Genscipt), and cloned into a mammalian expression vector pcDNA3.1(+) (Thermofisher Scientific). Constructs were purified using Endotoxin-free Qiagen Maxiprep kits and eluted in water. Female 6-week-old C57BL/6 mice (10 per group, strain 027, Charles River Laboratories) were rested one week after purchase and then administered a DNA vaccine three times at 3 week intervals. The DNA vaccines (30 µg of DNA in 30 µL PBS) were each injected into a hind leg thigh muscle, immediately followed by electroporation with a pair of electrode needles inserted into the muscle area surrounding the vaccine injection site. Electrical pulses were administered by using a BTX electroporation generator (catalog number ECM830; BTX Harvard Apparatus). Eight pulses of 106 V were delivered with a 20-ms pulse at 200-ms intervals. Serum samples were collected 2 weeks after the final vaccination for testing.

### Generation of VLPs in HEK 293 cells

To prepare VLP, Expi293™ human cells (ThermoFisher #A14527) were seeded at 3.6e6 cells/ml in 120 mL of Expi293™Expression medium (ThermoFisher#A1435101) in a 250 mL flask. In a 50 mL tube, 144 ug of plasmid expressing either HPV16 L1 and L2 (positive control p16ShLL; https://www.addgene.org/37320/), or HPV16 L1-mMUC16, L1-hMUC16 or L1-RG1 protein was added to 6 mL of Opti-MEM (Gibco #31985-070). In a separate 50 mL tube, 324 µL of ExpiFectamine (ThermoFisher #A14526) was added to 6 mL of Opti-MEM. After mixing and incubating at room temperature for 5 min, the diluted DNA was added to the diluted ExpiFectamine. The mixture was incubated at room temperature for 30 min and added to the cells. The transfected cells were incubated at 37 °C with shaking with 5% CO_2_ and moisture for 20 h before 0.6 mL of Enhancer 1 and 6 ml of Enhancer 2 were added. The incubation with shaking was continued for 72 h. Cells were then harvested into 50 mL tubes and pelleted at 1600 rpm at 4 °C. After discarding the culture medium, the cell pellet was resuspended and digested overnight at 37 °C in lysis buffer consisting of DPBS, 10mM MgCl_2_, 0.5% Brij58 (Sigma #5884-100G) and 0.2% Benzonase (Sigma #E1014-25kU). An aliquot was saved for Western blot analysis using the CamVir-1 monoclonal antibody. The lysate was cooled on ice for 5 min and salt concentration was adjusted to 850 mM with 0.17 volume of 5 M NaCl. The content was then transferred to siliconized microfuge tubes and incubated on ice for 15–20 min. The lysate was clarified by centrifugation at 10,000 rpm for 10 min at room temperature. 60% Optiprep (Sigma #D1556-250ML) density gradient was diluted to 27%, 33% and 39% with DPBS containing 0.8 M NaCl. The gradient was prepared in ultracentrifuge tubes (Beckman #344,060) with the 3 layers of Optiprep solutions. Each tube was filled with 1 ml of clarified cell lysate on top. The pellet was re-clarified twice and supernatant was placed on top of gradient layers in new centrifuge tubes. The gradients were centrifuged in a Beckman SV40.1 Ti rotor and Beckman L-80 ultracentrifuge at 40,000 rpm for 16 h at 16 °C. Following centrifugation, the VLP band was collected into siliconized 2 ml tubes and stored at -80 °C until use. The purity of fractions collected from gradient fractions was assessed via SDS-PAGE and Coomassie blue staining. VLP assembly was evaluated by transmission electron microscopy (TEM).

### Coupling of MUC16 peptides to L1 VLP and L1-RG1-VLPs

HPV16 L1-VLPs and HPV16-derived L1-RG1-VLPs were made and purified as described above. Prior to conjugation, the VLP preparations were dialyzed into reaction buffer (50 mM NaH_2_PO_4_, 500 mM NaCl, 2 mM EDTA, 0.01% (*v/v*) Tween 80, pH6.5). The disulfide bonds are reduced using TCEP (Tris 2-carboxyethyl phosphine, 51805-45-9, GoldBio) in a 10:1 (TCEP:VLP) molar ratio. After incubation in reaction buffer at room temperature for 1 h, mMUC16 or hMUC16 peptide with an N-terminal malemide modification (Genscript) was added in a 5:1 (peptide:HPV16VLP) or 10:1 (peptide:RG1 VLP) molar ratio. The mixture was incubated for another hour at room temperature with shaking and then dialyzed into storage buffer (500 mM NaCl, 0.01% (*v/v*) Tween 80 in phosphate-buffered saline, pH7) using Float-A-Lyzer®G-2 (G235062, MWCO 1000kD, Spectrum® Labs). After SDS-PAGE and Coomassie staining, the conjugated L1 was seen as a ladder of bands above the 55 kDa band of unconjugated capsid protein. The band immediately above the unconjugated band was considered to have one molecule of peptide attached per molecule of L1. The second conjugated band would have 2 peptides per L1, and so on. The percentage of peptide coupling was determined using ImageLab software (Biorad Laboratories), where percentage of the upper bands is the percentage of L1 conjugated with peptide. Integrity of the conjugated VLP was visualized using TEM.

### Vaccination with chemically-conjugated L1-RG1-VLP and L1-VLP via intramuscular injection

Eight-week-old C57BL/6 female mice (10 per group, strain 027, Charles River Laboratories) were vaccinated via intramuscular injection with the HPV16 L1-VLP or L1-RG1-VLP conjugated with mMUC16 or hMUC16 peptide. As controls for the VLPs conjugated with mMUC16 peptide, mice were also injected with each VLP containing an equivalent amount of loose mMUC16 peptide as was conjugated to the VLP, estimated based on one molecule of peptide conjugating to one molecule of L1. Each mouse received 10 µg of VLP (conjugated or with loose peptide), 50 µg of Alhydrogel® (vac-alu-250, Brenntag Biosector) in 100 µL PBS for three times 19 days apart. As negative controls, 5 mice were not vaccinated. Two weeks after the last injection, blood was collected and serum was separated and stored at -20^o^C.

### ELISA of serum antibody against MUC16 peptides and HPV16 L1-VLP

For biotinylated MUC16 ELISA, 96-well plates were coated with neutravidin (200ng/well) in 100 µL of PBS at 4 °C overnight. Neutravidin-coated plates were then incubated with biotinylated mouse MUC16, human MUC16 peptides, or RG1 epitopes (25 ng/well) in 100 µL of coating buffer (0.1 M Tris, 0.1 M NaCl, 0.1% Tween-20, pH 7.4) at 4 °C overnight. For the HPV16 L1-VLP ELISA, 96-well plates were coated with HPV16 L1-VLP (80 ng/well) in 100 µL of PBS at 4 °C overnight. The next day, plates were blocked with PBS containing 4% skim milk (232,100, BD Difco™) and 0.2% Tween® 20 (P7949, Sigma-Aldrich) for 1.5 h at room temperature and subsequently incubated with 2-fold serially diluted sera, 8 times beginning at 1:100 in blocking buffer for 1 h at room temp, followed by incubation with an HRP-conjugated goat anti-mouse IgG secondary antibody (A4416, Sigma Aldrich) at 1:5000 dilution for VLP in blocking buffer for 1 h at room temperature. HRP substrate (KPL#50-76-03, GE Healthcare) was used for development and the reaction was stopped with 0.36 N of H_2_SO_4_ (made from a 2 N solution, C748W85, Thomas Scientific) after a 30 min incubation in the dark at room temp. Absorbance was read at dual wavelengths of 405 and 620 nm. Background absorbance at 620 nm was subtracted from that at 405 nm before data points were plotted in GraphPad Prism 7 software program. Antibody isotyping studies utilized the hMUC16 peptide ELISA but with the Mouse typer isotyping panel (Biorad #1,722,055) diluted 1:3 in blocking buffer as secondary antibody followed by HRP-conjugated goat anti-rabbit IgG secondary antibody.

### Flow cytometry analysis

OVCAR3 and SKOV3 cells (ATCC) cultured per ATCC recommendations were first treated with Fc Block (2.4G2 antibody 1:50, Biolegend) for 30 min at 4 °C prior to antibody staining. Cells were then stained with sera from vaccinated mice diluted in 1:50 in staining buffer [PBS with 0.2% bovine serum albumin (BSA)] at 4 °C for 30 min, washed twice with staining buffer and then stained with PE-conjugated goat anti-mouse IgG antibody (1:200 BioLegend, San Diego, CA) at 4 °C for 30 min. Cells were then washed twice with staining buffer and resuspended in staining buffer for flow analysis. Single staining controls of ultracomp ebeads (Thermo Fisher Scientific, Waltham, MA) were used to set a compensation matrix for each experiment. Data collection was done on a 13-color Beckman Coulter CytoFLEX S, and analysis was done with FlowJo 10.4 software (FlowJo LLC).

### Statistical analysis

All data are expressed as means ± standard error of the mean (S.E.M). The statistical significance was determined by one-way ANOVA with Tukey-Kramer multiple comparisons or Student’s t-test using Prism 9 software (GraphPad, CA, USA). In all circumstances, *p*-values ≤ 0.05 were considered significant (*, *p* < 0.05; **, *p* < 0.01; ***, *p* < 0.001; ****, *p* < 0.0001).

## Data Availability

The datasets used and/or analyzed during the current study are available from the corresponding author on reasonable request.

## Abbreviations

AA: Amino acid
ADCC: Antibody-dependent cellular cytotoxicity
BSA: Bovine serum albumin
CDC: Complement-dependent cytotoxicity
HPV: Human papillomavirus
VLP: Virus-like particles
